# A Mixed-Methods Examination of Inpatient Breastfeeding Education Using a Human Factors Perspective

**DOI:** 10.1089/bfm.2021.0087

**Published:** 2021-12-07

**Authors:** Elizabeth Lerner Papautsky, Mary Dawn Koenig

**Affiliations:** ^1^ Department of Biomedical and Health Information Sciences, College of Applied Health Sciences, University of Illinois Chicago, Chicago, Illinois, USA.; ^2^ Department Human Development Nursing Science, University of Illinois Chicago, Chicago, Illinois, USA.

**Keywords:** patient-provider communication, education, inpatient, breastfeeding, mixed-methods

## Abstract

***Objective:*** The objective of this study was to examine postpartum, inpatient mother-lactation educator (LE) breastfeeding education, resulting perceptions, and patient-reported worries and outcomes. In the breastfeeding literature, there is inadequate insight into the mother-LE relationship, and specifically, the extent to which contextual factors are elicited and information is tailored accordingly. In this study, we were specifically interested in maternal contextual factors.

***Materials and Methods:*** Using a mixed methods approach, we (1) captured 20 postpartum, inpatient mother-LE breastfeeding education sessions and analyzed them for the presence of maternal contextual factors, (2) administered separate perception questions to mothers and LEs, and (3) conducted 13 follow-up interviews with mothers after being discharged from the hospital.

***Results:*** Inpatient breastfeeding education is delivered in dynamic and busy clinical settings, characterized by potential distractions such as delivery of medical care. Maternal contextual factors are infrequently elicited during the education. Although both LEs and mothers rate the sessions positively, potential gaps remain as highlighted by the analyses of semistructured interviews with mothers.

***Conclusion:*** Human factors perspective, theories, and methods are relevant to the characterization of facilitators and barriers of current breastfeeding education, as well as to the development of interventions to support the delivery of human-centered, effective, and timely breastfeeding education.

## Introduction

Despite a robust literature demonstrating the multiple benefits of breastfeeding on both maternal* and infant health,^1–4^ continuation of breastfeeding beyond the immediate postpartum period remains a challenge, especially among low-income communities.^5^ Although most individuals intend to breastfeed and initiate breastfeeding at birth (∼85%), many do not continue to breastfeed beyond the immediate postpartum period, and almost a quarter of infants are supplemented with infant formula before 2 days of age.^5^ In fact, only one-third of US infants are still breastfed at 1 year^6^ despite the American Academy of Pediatrics (AAP) recommendations.^7^ In the United States, disparities in breastfeeding persist among certain subpopulations. Despite similar prenatal breastfeeding intention rates across subpopulations,^8^ women who identify as non-Hispanic white, non-Hispanic black, and Hispanic initiate breastfeeding at rates of 86.7%, 73.7%, and 84.1%, respectively; continue exclusive breastfeeding through 6 months at rates of 52.4%, 38.7%, and 41.5%, respectively; and breastfeed through 12 months at rates of 28.7%, 21.2%, and 21.5%, respectively.^5,9^

Research that addresses barriers to continued breastfeeding has focused on identifying, describing, and mitigating barriers, such as socioeconomic factors and social support.^10,11^ A qualitative study suggested that the decision around breastfeeding initiation in black mothers is a function of a multitude of contextual factors (e.g., role of others, communities, information sources, providers, and health organizations).^12^ Thus, maternal contextual factors (e.g., characteristics, life or situational circumstances) both within and beyond the immediate postpartum period can negatively impact breastfeeding duration.^13^ Women make sense of and experience breastfeeding support differently, and their experience is heavily dependent on their cultural context, prior experiences, and desires.^14^ Tailoring of breastfeeding education with consideration to maternal contextual factors is essential for the promotion of continued breastfeeding.^14^ High-quality antenatal and postpartum care and education play a critical role in breastfeeding success.^15^ Hospitals that achieve Baby-Friendly^®^^45^ designation and follow the 10 steps to successful breastfeeding have higher breastfeeding rates.^16–18^ Several of the 10 steps (which include breastfeeding within 1 hour of birth,^19^ allowing mothers and infants to remain together 24 hours a day,^20^ and no food or drink other than breast milk unless medically indicated^21,22^) have demonstrated increased success with the continuation of breastfeeding beyond the immediate postpartum. However, many women do not receive the high-quality care and education that they need.^23^ For many lactation educators (LEs), this is not due to a lack of professional interest, but due to increasing demands with fewer resources. In addition, there is no current systematic approach to assess the effectiveness of patient breastfeeding education. Because of these constraints, breastfeeding education often takes a “one size fits all” approach.

The field of human factors is focused on ensuring information is tailored to the recipient based on context in support of safety and quality. In the last decade, human factors science has become integral in patient safety research and operations.^24^ Similarly, the concept of “contextualized care” (concerned with adapting care to individual patient context) has been recently popularized to inform more appropriate care and planning.^25^ Consideration for individual patient context, preferences, and attitudes is at the core of patient-centered care and has been associated with satisfaction and positive outcomes for patients and their families. By gathering relevant contextual information from patients, particularly about life and social constraints, members of the health care team are better able to tailor treatment plans accordingly to support positive outcomes. Similarly, in this study, we sought to tap into patient–provider interactions to understand how breastfeeding education is delivered.

There is a dearth of research that focuses on patient-provider breastfeeding education, and specifically the extent to which information is tailored to the goals, abilities, and situations of individual mothers. Research has generally focused on educational approach, and contextual factors have been addressed only as secondary outcomes in studies.^26–29^ Specifically, the number of visits, visit duration, and education content are evaluated.^26–30^ In fact, research has demonstrated that breastfeeding interventions should include not only the mother but also their partners and broader networks.^31^ However, breastfeeding education and patient-provider communication around breastfeeding have not been examined from the human factors perspective, and it is unknown to what extent breastfeeding education is sensitive to the individual mother's context.

### Purpose

The main objective of this research study was to examine—using a human factors perspective—postpartum mother-provider breastfeeding education and subsequent patient attitudes, behaviors, and outcomes. Please note that in this article, we will be using the term LEs for those providing the lactation service and the term *education* to refer to an educational session that takes place between mothers and LEs. Using a mixed-methods approach, we (1) captured postpartum mother-provider breastfeeding education sessions at the hospital and analyzed them for the presence of contextual factors; (2) administered separate perception questionnaires with mothers and providers, along with an interview with the mothers; and (3) conducted a follow-up interview with mothers to capture attitudes, behaviors, and breastfeeding outcomes after being discharged from hospital. We hypothesized that early postpartum breastfeeding education is not aimed at eliciting maternal context.

## Materials and Methods

This research complies with the American Psychological Association Code of Ethics and was approved by the Institutional Review Board at University of Illinois Chicago (Protocol #2017-1383).

### Setting

The study took place in a single clinical setting within the Postpartum/Antepartum Stepdown unit in a large, inner city academic hospital, providing care to primarily black and Hispanic low-income communities. At the time of the study (2018), 2,238 infants were delivered in the hospital and it did not have a Baby-Friendly designation. A breastfeeding policy was in effect with the objectives of (1) providing education to families on the benefits of breastfeeding and breast milk; (2) supporting recommendations from relevant agencies such as AAP, Academy of Breastfeeding Medicine, and others; (3) complying with the requirement that hospitals in this state adopt a policy that promotes breastfeeding; and (4) working toward achieving specific breastfeeding target rates. The LEs were directed to cover topics associated with hormones, feeding frequency, tracking output, cues for swallowing, watching for engorgement, and others. Six LEs were on staff, delivering breastfeeding support services on both the Postpartum/Antepartum Stepdown unit and a separate neonatal intensive care unit (NICU) to mothers experiencing breastfeeding challenges.

### Participants

In 2018, we captured 20 education sessions between mothers and LEs to achieve saturation for qualitative analyses. Participants included 20 mothers within 2 days postpartum and 6 LEs. Thirteen mothers were followed up at 1–2 weeks postdischarge from the hospital.

#### LE recruitment

The lead LE on the unit assisted with recruitment of the staff LEs. In a private location, a researcher provided each LE with a description of the study and, if interested, asked them to read and sign the consent from. In the consent form, LEs were asked to indicate whether they would consent to being audio-recorded. All LEs consented to participate in the study and to be audio-recorded.

#### Patient (mother) recruitment

On a data collection day, a researcher arrived on the unit and an LE supplied the researcher with the room numbers of mothers that were scheduled to receive breastfeeding education during that shift. Eligible mothers consisted of those having expressed interest in breastfeeding, recovering on the unit after a nontraumatic birth to a single, term baby. A clinician had previously asked each mother about their interest in breastfeeding and documented their answer accordingly in the patient medical record. Breastfeeding education was only provided by LEs to those mothers who expressed an interest in breastfeeding. Mothers of babies in the NICU and/or of multiples were excluded. Data collection began at 8 AM. The researcher visited the rooms of mothers based on the time elapsed since giving birth, starting with the longest. The researcher provided a verbal study description and asked about the mother's interest in the study. If a mother was interested, the researcher asked them to read and sign the consent form. In the consent form, mothers were asked to indicate whether they would consent to being audio-recorded. All mothers agreed to be audio-recorded. The researcher returned when the LE came to the patient's room to provide breastfeeding education.

### Materials

To compare perceptions of the education, specifically for this study, we developed and administered three matching quantitative perception questions for the LE and the mother. We also developed interview guides for semistructured interviews with the mother after the inpatient hospital education and 1–2 weeks postdischarge.

### Procedure

During the breastfeeding education, the researcher positioned themselves in a chair located in the room to observe and audio-record. In addition, the researcher took real-time notes focused on describing the setting, including the location of the baby in relationship to the mother (couplet status), presence of others (e.g., the baby's father), and events that occurred during the education in addition to the information exchange. We termed these factors *education setting descriptors*.

Immediately following the education, we administered the perception questions to the LE and the mother separately and consecutively (5 minutes each). Then the mother participated in a semistructured interview (∼25 minutes). At completion of the inpatient components of data collection, the researcher debriefed and compensated the mother in cash. We made three attempts to contact all 20 mothers via phone and text 1–2 weeks postdischarge from the hospital to participate in a 15-minute follow-up phone interview (Time 2). Thirteen mothers were reached and agreed to participate. At the completion of Time 2, all participating mothers were debriefed, and compensation was mailed to their home address.

### Analysis

We used Microsoft Word and Excel as well as Dedoose^®^, a web application for qualitative data analysis.

#### Education

Each observation of an education session yielded notes and audio recordings. We noted each recording's duration in a spreadsheet. We then manually de-identified (by removing any mentions of mother's or baby's names) the audio recordings using Audacity^®^ and submitted them to a transcription service. We systematically reviewed and edited transcripts for accuracy.

Using researcher notes, we documented education setting descriptors in a spreadsheet and calculated frequencies proportions. Using the transcripts, we calculated descriptive statistics (means, standard deviations, and ranges) for total words, total words per LE, total words per mother, questions asked by LE, and questions asked by mother. We identified and calculated the proportion of the sessions in which mothers' contextual factors were elicited by the LE, delivered by the mother, or not mentioned.

#### Education perception questions

We calculated means and standard deviations of the responses to the quantitative questions for mothers and LEs.

#### Semistructured interviews (Time 1 and Time 2)

We conducted an analysis of the interviews with a focus on (1) mothers' worries and challenges related to breastfeeding, (2) questions related to breastfeeding not asked the mothers, and (3) mothers' reporting of memorable information regarding the session. For Time 2, we identified mother-reported breastfeeding and lactation outcomes.

## Results

Below, we present the findings associated with subjective and objective measures and perspectives of mothers and LEs. The sections are as follows: participants, education setting, education description (quantitative descriptors of time, words, and inquiries), education content (maternal contextual factors), education perception (quantitative reporting of mother and LE responses), and patient-reported worries and outcomes.

### Participants

Demographic characteristics of the participants are reported in Table 1. We were unable to obtain insurance information for 10 of the mothers; of the other 10, 50% (*n* = 5) had Medicaid. LEs had a wide range of experience in their role and in health care (4 months to 9 years), although all had bachelor's degrees. All LEs were female. All were either International Board of Lactation Consultant Examiners^®^ (IBLCE^®^) certified or in the process of certification and were providing breastfeeding education individually. Some had additional lactation training (e.g., La Leche League). There were up to two LEs on the shifts when data collection took place. Mothers were provided with a $25 incentive at Time 1 and at Time 2 for a total of $50. LEs did not receive an incentive.

**Table 1.** Mothers' and Lactation Educators' Demographic Characteristics

Mothers (*N* = 20)		LEs (*N* = 6)	
Age	18–35 range	Age	30–65 range
Black	14 (70%)	Black	1 (17%)
Hispanic	6 (30%)	White	5 (83%)
Employed	12 (60%)	Certification	International Board Certified Lactation Consultant (completed or in progress)
Unemployed	7 (35%)	Experience in health care	4 months to 9 years
High school student	1 (5%)	Experience in LE role	4 months to 9 years
Income <$30,000/year	11 (55%)	Education	Bachelor of Science

LEs, lactation educators.

### Education setting

Based on the notes captured in real-time, we characterized the setting by couplet status, others present, environmental distractions, and maternal distractions. We calculated proportions accordingly, reported in Table 2. The baby was not always physically present with the mother during the education. In only 60% (*n* = 12) of sessions was the baby physically with the mother, and in 2 of those 12 sessions, the baby was either absent at the outset or removed during the education. There were multiple events that took place during the education, including the delivery of medical care unrelated to breastfeeding (*n* = 4) and meals (*n* = 7). In multiple cases (*n* = 4), the mother was talking or texting on a cell phone while the LE was speaking. We also noted the presence of others (baby's father, mother's mother, and so on), which was the case in 75% (*n* = 15) of the sessions. During the breastfeeding education if others were present, they were not engaged by the LE in most sessions.

**Table 2.** Descriptors and Corresponding Proportions of Breastfeeding Education Setting (*N* = 20 Mothers; *N* = 6 Lactation Educators)

	Description	% Sessions
Couplet status	Baby with mother	60
	Baby in bassinet	30
	Baby with other	20
	Baby absent	5
	Baby removed during session	5
	*Note: couplet status may have changed throughout session*	
Others present	Any individual	75
	Baby's father	45
	Mother's mother	15
	Mother-in-law	0
	Mother's sibling(s)	5
	Roommate/other	10
Environmental events	Meal delivery	35
	Delivery of medical care	20
Maternal distractions	Phone call/text receiving/responding	20

### Education description

Our findings, reported in Table 3, demonstrate the variability of the quantitative measures of the education. There is a wide range for education duration and total number of words exchanged. To gain insight about communication patterns associated with common grounding, we examined the number of inquiries. LEs did most of the talking and conducted most of the inquiries (*M* = 11.90, *SD* = 5.10). Mothers made few inquiries; in some cases, none, with a maximum of 10. Given our small sample size, especially for LEs, we were unable to examine factors associated with individual differences in communication.

**Table 3.** Quantitative Description of Breastfeeding Education (*N* = 20 Mothers; *N* = 6 Lactation Educators)

	Encounter duration (minutes)	Total words	Total words (LEs)	Total words (mother)	Total inquiries	Inquiries by LEs	Inquiries by mother
Mean	15.6	2357.3	1997.8	359.6	13.8	11.9	2.0
SD	7.0	923.8	751.9	249.1	6.5	5.1	2.4
Range	4.03–28.18	653–3,882	636–3,226	17–989	6–29	5–23	0–10

SD, standard deviation.

### Education content

As represented in Table 4, in the majority of sessions, contextual factors of work (i.e., the mother's timeline of going back to work) were not shared in 75% of cases, work environment in 95%, and general support system in 80%. Discussion of children primarily took place in the context of previous breastfeeding experiences, rather than as a potential barrier for future breastfeeding success. Also, a latching demonstration was not initiated in 40% (*n* = 8) of sessions. In some of these cases, the baby was either absent or sleeping, but that was not always the case. None of the sessions included an up-front question about the mother's goals as a function of her context.

**Table 4.** Proportion of Sessions That Included Mother's Contextual Factors (*N* = 20 Mothers; *N* = 6 Lactation Educators)

Mother's contextual factors	LEs elicited (%)	Mother delivered unprompted (%)	Not shared (%)
Other children	50	30	20
Work	10	15	75
Work environment	0	5	95
Support system	15	5	80

### Education perception

LEs and mothers reported high level of agreement with questions addressing patient understanding and active involvement in the conversation, as well as feeling good about the conversation. Means and standard deviations are reported in Table 5.

**Table 5.** Means and Standard Deviations of Mothers' and Lactation Educators' Education Perception Ratings (on a Scale of 1 [Completely Disagree] to 7 [Completely Agree])

Posteducation questions for LE	Mean	SD	Posteducation questions for mother	Mean	SD
Patient understood what I told her	6.47	0.70	I understand what the lactation educator just told me	7.00	0.00
Patient was actively involved in the conversation	6.42	1.12	I felt like I was actively involved in my conversation with the lactation educator	6.48	0.23
I feel good about my conversation with the patient	6.30	0.66	I feel good about my conversation with the lactation educator.	7.00	0.00

### Patient-reported worries and outcomes

We analyzed semistructured interviews with mothers at Time 1 and Time 2. At Time 1, the top three worries reported by mothers included underfeeding or overfeeding (35%, *n* = 7), milk supply and production (15%, *n* = 3), and latching (35%, *n* = 7). At Time 2, the most frequently reported challenges were latching (15%, *n* = 2) and feeding schedule (15%, *n* = 2). At Time 1, almost all mothers (90%, *n* = 18) reported that there were no questions they wished they had asked the LE but did not. The two that responded in the affirmative wished they had asked about partner help and pumping. Time 2 interviews revealed a different pattern of responses regarding this question, with 62% (*n* = 8) reporting in the affirmative and 31% (*n* = 4) in the negative (*n* = 1 did not provide a response). Specific topics (came up once) included latching, engorgement, milk storage, timeline of a breastfeeding session, painful breasts, milk formation, and taking medications. At Time 2, only one mother was breastfeeding directly exclusively, four mothers (33%) were breastfeeding and pumping, five mothers (42%) were using breast milk and formula, and two were using formula only. All but one mother reported the hospital-provided breastfeeding and lactation information packet to be a helpful source of information after discharge. We also examined responses to the following question asked at Time 2: *What did the LE say that stood out to you and was most useful?* Examples of responses included the following, among others:

- “Nipples should be shaped like a lipstick when baby is latching on” (note: this may indicate a misunderstanding on the part of the mother)
- “Just give it to him like you're feeding him a sub sandwich….”
- “How to position the breast when latching—how to hold the breast and position the nipple…”
- “Changing positions was very helpful. Positioning of nipple. Pumping brings nipple out so baby will latch more of it.”
- “Massaging breast to get the rest of the milk out when pumping.”
- “Nothing.”
- “Helped explain breastfeeding process shouldn't hurt or be painful.”

## Discussion

**FIG. 1.**
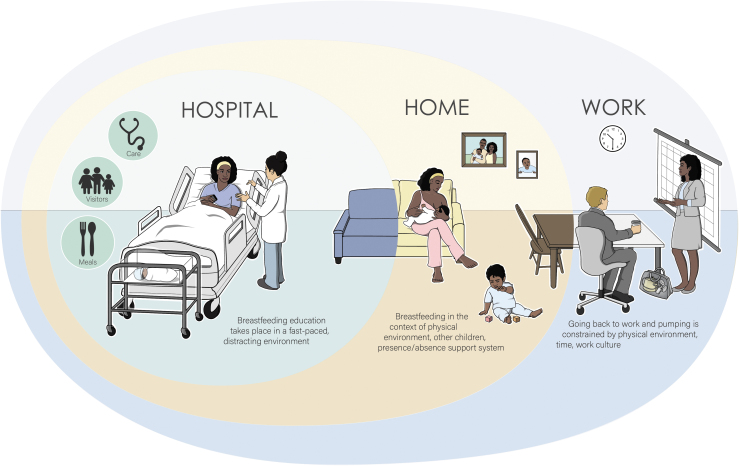
Representation of breastfeeding and lactation in context (hospital, home, work, and world). (©2019, Casey Garr, printed with permission from Garr).

This is the first study to use a human factors approach considering the individual mother's contextual factors to examine postpartum mother-provider breastfeeding education and subsequent patient attitudes, behaviors, and outcomes. We present data regarding breastfeeding and lactation in context—hospital to home to work (e.g., in the world), across time, and we illustrate the contextual factors at play that may serve as barriers and facilitators in breastfeeding outcomes (Fig. 1). Given their potential to impact inpatient breastfeeding education, a multitude of factors characterized in our study, including the absence of the infant during the education session and the infrequent discussion of work-related and childcare responsibilities postdischarge, were identified as critical to the success of continued breastfeeding.

Systems of care for mothers needing breastfeeding support are often fragmented and lack coordination of care and effective service delivery.^32^ Specifically, many hospital protocols and busy postpartum units suffer from challenges and drive practices that undermine breastfeeding and inhibit coordination.^33^ Reflected in the education setting analysis, the situational factors associated with the status of the mother and baby in relationship to each other, the presence of other individuals, and other events taking place need to be considered and incorporated into inpatient breastfeeding education given their role in facilitating or hindering education. For instance, a hands-on breastfeeding demonstration is not possible if the baby is not present. Demonstration is important given that breastfeeding is a perceptual task^34,35^ as it relies on visual, tactile, haptic, and auditory cues. The delivery of a meal or medical care can be distracting to the mother and/or the LE by taking attention away from the conversation.^36^ Hands-on demonstration requires the LE to be present and available to the mother at multiple time points throughout their hospital stay, which is not always possible due to staffing and budget considerations. Being short-staffed or otherwise time-constrained is a common problem for LEs, leading to role strain, nonevidence-based practices, and poor quality of services and care.^32^ Budget cuts and lack of support from administrators impact staffing rations for LEs and have direct consequences for service delivery.^37,38^ Stable and good-sized professional staff is indispensable for the implementation and promotion of evidenced-based practices related to breastfeeding.

Word and inquiry counts provide insight into the balance of the conversation, as well as verbal engagement of the mother during the session. Papautsky and Shalin^39^ analyzed inquiries in a laboratory team planning task. They found that when shared information was absent or limited, inquiries helped to achieve common ground. The sparsity of questions asked by mothers suggests that they did not actively work to establish such common ground, perhaps because mothers did not yet know what else they needed to know (as information needs would only emerge as a function of experience with breastfeeding attempts after discharge).

Maternal contextual factors, particularly around caring for other children, returning to work, work environment, and support system were infrequently discussed. Returning to work is one of the main barriers for breastfeeding in the United States.^40^ Characterization of support systems (or lack thereof) and children, and additional factors that have not yet been identified, need systematic investigation. Given that consideration for patient context, preferences, and attitudes is at the core of patient-centered care and is associated with satisfaction and positive outcomes in patients suffering from illness,^41^ more deliberate attention on these topics is needed in breastfeeding education to support expectation management for transitioning to and between settings. Particularly, eliciting contextual factors can support the tailoring of breastfeeding information to the needs of each mother and also help to anticipate and prepare for future potential challenges. An opportunity for discussion of contextual factors can be created by an up-front elicitation and unpacking of mothers' goals.

Education perception ratings were high and similar across LEs and mothers. However, they were collected at a time when future breastfeeding challenges were not yet known. Therefore, neither mothers nor LEs were in a position to make judgments on the quality of the education, but rather on the conversation. Coupled with a shift in information needs and worries between Time 1 and 2, these findings highlight the need for longitudinal examination of acquisition of breastfeeding knowledge and experience to inform how breastfeeding can best be supported on the front-end. Although Time 2 interviews did not reveal the maternal contextual factors, perhaps they were conducted too soon after discharge, while mothers were still in a time of adjustment to the new baby at home, potentially receiving more support than they might later and with less concern regarding work.

Finally, inpatient breastfeeding education is a specific interaction with the health care system at a point in time, but it must be considered as embedded in the whole of the patient journey. Our breastfeeding and lactation in context visualization (Fig. 1) is specifically in line with Systems Engineering Initiative for Patient Safety (SEIPS) 3.0, which calls for the need to examine patient work (in this case, the mother's work associated with lactation and breastfeeding) as spanning space and time.^42^ Specifically, SEIPS 3.0 is a human factors health care model that highlights the multitude of sociotechnical and individual factors that need to be considered when examining the work of clinicians, patients, and families.

### Limitations

Although our study is limited by a small sample size and single-site location that did not have a Baby-Friendly designation, our study identifies potential gaps and opportunities for improvement in mother-provider breastfeeding education. As with all research that takes place in real-world environments, a systems perspective is necessary.^43^ Not unlike other clinicians and staff, LEs conduct their work in complex, busy, and time-sensitive settings where medical care takes precedence. Mothers may be tired, overwhelmed, and distracted. These factors undoubtedly play a role in the timeliness and effectiveness of the education.

It is critical to note that this study would not have occurred had it not been for the ease of transition into the unit as a research team that was supported and welcomed by the lead LE and the nursing director. Our research team was accepted, and provided with access to the unit, LEs, and patients. Through our interactions with the LEs outside of the data collection, we learned that they were genuinely concerned with supporting the mothers, including ensuring that mothers got timely and needed information. At the completion of the study, the PI was approached for feedback that could inform improvements to breastfeeding education on the unit.

## Conclusions

Our study is novel in examining inpatient breastfeeding education from the human factors perspective. Our findings highlight the need to further investigate the role of considering contextual factors, training and education modality, and learning assessment during a critical time in establishing milk supply. Despite study limitations of a small sample size and a single site, our findings highlight the need to further investigate this problem space. The area of breastfeeding and lactation education could benefit from a human factors perspective, a discipline that is focused on tailoring solutions to user needs.^44^ Further, such investigation is particularly critical as there are currently no Baby-Friendly guidelines associated with tailoring information to the needs of individual patients or specific patient populations.

Thus, understanding maternal context and goals outside of the specific interaction and tailoring information to that context is consistent with human factors objectives of developing human-centered solutions. In addition, human factors literature on topics such as training development and evaluation may be particularly relevant to the tasks of lactation and breastfeeding given cognitive and perceptual task complexity. During their hospital stay, postpartum women should be provided with breastfeeding education that is effective, use appropriate modalities (e.g., include demonstrations for perceptual task components), and patient-centered—tailored to mother's unique life context, goals, and abilities. We suggest that these aims are in complement to the current *Baby-Friendly Hospital Initiative: Guidelines and Evaluation Criteria for Facilities Seeking and Sustaining Baby Friendly Designation*, 2019.^45^

### Implications and recommendations

Findings from our study have the potential to inform the development of human-centered training and support interventions for inpatient breastfeeding education to ensure that it accounts for maternal contextual factors, to improve breastfeeding outcomes. We suggest the following next steps as recommendations for future research directions and operational interventions:

- Examine maternal life context and knowledge needs/requirements prenatally, postpartum inpatient, as well as following discharge, longitudinally.
- Explore relationships between prenatal and postpartum, inpatient breastfeeding education, and outcomes.
- Tailor breastfeeding education based on maternal context.
- Prioritize delivery of inpatient breastfeeding education in the context of environmental and institutional constraints (e.g., mitigate environmental distractions).
- Create and optimize opportunities for patient-provider teaming in education, information sharing, and planning in service of better breastfeeding outcomes.
- Include hands-on practice (i.e., demonstration) as part of education given that breastfeeding is a complex perceptual task.
- Explore benefits of different types of LE support.
- Ensure active presence of both mother and baby during the breastfeeding educational session (as well as significant others and family members as support).
